# An online solution focused brief therapy for adolescent anxiety during the novel coronavirus disease (COVID-19) pandemic: a structured summary of a study protocol for a randomised controlled trial

**DOI:** 10.1186/s13063-020-04355-6

**Published:** 2020-05-13

**Authors:** Shitao Chen

**Affiliations:** Beijing Normal University, Faculty of Psychology, Beijing, China

**Keywords:** COVID-19, Randomized controlled trial, Protocol, Solution focused brief therapy, Adolescents, Anxiety

## Abstract

**Objectives:**

This study aims to assess the effectiveness of delivering Solution Focused Brief Therapy (SFBT) through telecommunication with a group of adolescents who present anxiety symptoms during the COVID-19 outbreak. We hypothesize that participants who are randomly assigned to receive 2–4 sessions of Solution Focused Brief Therapy would have better clinical outcomes than participants who are in the waitlist group. We additionally hypothesized that using SFBT can also change participants’ depression levels and their coping strategies in dealing with distress during the COVID-19 pandemic.

**Trial design:**

This study employs a randomized delayed crossover open label controlled trial in adolescents who are presenting anxiety symptoms during the COVID-19 outbreak. Participants who meet the enrollment criteria stated below will be invited to participate in this study through telecommunication. Those accepting will be randomly allocated to the intervention group or waitlist group.

**Participants:**

The inclusion criteria for participants are:

1. Adolescents between 11 and 18 years old who are currently in grade 7–12
2. Manifesting anxiety symptoms during the COVID-19 outbreak and Generalized Anxiety Disorder (GAD) 7 ≥ 10
3. Having a legal guardian who has signed the informed consent and an adolescent willing to participate in this study
4. Have a stable internet condition and a quiet space for receiving internet-based counseling

The exclusion criteria are:

1. Reporting suicidal ideation or plan during the past 2 weeks
2. Receiving counseling service elsewhere
3. Taking psychiatric medication
4. Having another severe mental health diagnosis (e.g., bipolar disorder, psychosis)
5. Unwilling to have legal guardian sign the informed consent or participate in this study

All data were collected through a Chinese online survey tool called “Wen Juan Xing” (https://www.wjx.cn/) from the Faculty of Psychology at Beijing Normal University.

## Intervention and comparator

The intervention group will receive Solution Focused Brief Therapy 2–4 times within a 2 week period through telecommunication (e.g., using a platform such as Zoom). The comparators will be assigned to the waitlist group. They will also receive 2–4 sessions of counseling service after the 1 month follow-up data is collected.

## Main outcomes

The primary outcome measures of the study are intended to assess the difference in adolescents’ anxiety levels when comparing the SFBT group and the waitlist group. Level of anxiety will be measured by two measurements: GAD-7 and STAI-Y (C). Follow up 2 weeks and 1 month.

Baseline scores (GAD-7, STAI-Y(C), PHQ-9, CSS) will be collected from all participants and their legal guardian (Spence Children’s Anxiety Scale-Parent report, Patient Health Questionnaire − 9) before beginning treatment. After 2 weeks’ treatment, participants from both the intervention group and waitlist group will complete the assessments (GAD-7, STAI-Y(C), PHQ-9, CSS, CSQ-8), and their legal guardian will also complete several assessments (SCAS-Parent report, PHQ-9). After 1 month of treatment, data will be collected again.

Note: GAD-7 (Generalized Anxiety Disorder-7); STAI-Y(C) (State-Trait Anxiety Inventory); PHQ-9 (Patient Health Questionnaire-9); CSS (Coping Style Scale for Secondary School Students); CSQ-8 (Client Satisfaction Questionnaire-8), SCAS (Spence Children’s Anxiety Scale).

## Randomization

The Random Number Generator, an internet-based randomization tool, is used to assign participants to the intervention group and waitlist group. The allocation ratio is 1:1.

## Blinding (masking)

Participants will be informed about the group assignment due to the nature of the study design. The research assistant who will have access to the outcome data is blinded to group assignment.

## Numbers to be randomized (sample size)

The study plans to enroll a total of 76 participants. Thirty-eight participants are to be randomized to each group.

## Trial status

The Protocol version number is 02, with ethical approval number 202003130012. The recruitment is ongoing. It started on March 20th, and we estimate it will finish by the 30 June 2020.

## Trial registration

The trial was registered with the Chinese Clinical Trial Registry on March 20th, 2020. Registration number is ChiCTR2000030989.

## Full protocol

The full protocol is attached as an additional file, accessible from the Trials website (Additional file 1). In the interest of expediting dissemination of this material, the familiar formatting has been eliminated; this letter serves as a summary of the key elements of the full protocol.

## Supplementary information

**Additional file 1:** Full-study protocol in Chinese.

## Data Availability

SC and JH will have access to the final trial dataset. The dataset will be available from the author upon reasonable request. The contact email address is chenshitao@bnu.edu.cn

