# Supplementary material for: An online solution focused brief therapy for adolescent anxiety during the novel coronavirus disease (COVID-19) pandemic: a structured summary of a study protocol for a randomised controlled trial
Source: Trials. 2020 May 13;21:402. doi: 10.1186/s13063-020-04355-6 (PMC7218649; doi:10.1186/s13063-020-04355-6)
Supplement: Supplementary file 1 — Additional file 1: Full-study protocol in Chinese. [file 13063_2020_4355_MOESM1_ESM.docx]

作者：陈师韬

chenshitao@bnu.edu.cn

使用网络短程焦点咨询干预新冠肺炎疫情下的青少年焦虑症状：一项采用平行对照组，数据分析员单盲的随机对照实验

研究方案

**研究方案**

**项目基本信息**

1. 研究项目标题：

使用网络短程焦点咨询干预新冠肺炎疫情下的青少年焦虑症状：一项采用平行对照组，数据分析员单盲的随机对照实验

1. 研究的临床注册号：

本研究已于2020年3月20日通过中国临床试验注册中心的注册，注册号为ChiCTR2000030989

1. 版本编号： 02
   1. 01版本对研究的设计进行详细的描述
   2. 02版本增加了临床注册的具体版本号码
2. 基金支持：

本研究得到北京师范大学教育基金会支持。该基金会提供的资金可用于被试费发放、咨询师的劳务费发放、针对咨询师的培训和督导费用、以及部分的数据分析费用。

1. 角色和责任：
   1. 本研究方案的主要策划者是陈师韬。她提出了该项研究计划并且招募团队来进行方案的落实。具体包括研究方案的写作、研究人员的培训、被试的审核、对咨询师的培训、咨询过程的督导、以及对项目进展的把握；陈师韬还会是整个研究论文的主要写作者。陈润森为项目的外部咨询专家，为研究的设计、数据的分析以及项目进展中的问题提供了参考的建议，有利的指导了该项目的进展。项目的主要运营科研助手为郭兰心和钟颖。两位工作人员主要负责来访者的招募以及和来访者的沟通协调。项目的初筛评估人员为刘子慧、陈宇希、余泳蓝、郭兰心、钟颖、伍斐、吴瑾，她们主要负责对符合初筛条件的来访者进行ADIS的访谈，并且进一步明确来访者是否符合入组标准。项目的咨询师为毕文秀、陈沛昱、冯晓莹、袁泉、王瑾，她们经过主要负责人陈师韬的培训，为项目中进入干预组的来访者提供短程焦点取向的2-4次心理咨询服务。另外，赫嘉番主要负责整个项目中的问卷设计、问卷数据收集，以及后期的数据分析工作。
   2. 本次研究的资助方为：北京师范大学教育基金会。

基金会法人：张凯

基金会联络人：杨艺

联系方式：电话（010）58800780或者（010）58802083；

邮箱：[bnuef@bnu.edu.cn](mailto:bnuef@bnu.edu.cn)

地址：北京，海淀区，新街口外大街19号

- 1. 本研究的资助方只对研究提供资金支持，不参与研究的设计、被试的招募、干预的提供、数据的分析等具体内容，也不对文章的发表有任何决定性的权利。
  2. 不同团队的责任：

团队1：项目主要负责人和外部专家主要负责的内容包括：

设计和落实该研究项目

准备该研究项目同时做不同的完善

准备项目的不同流程设计

组建不同的团队分工

准备个案报告表

发表文章

团队2：项目运营团队的负责内容包括：

来访者招募海报的设计

来访者的具体招募和筛选

完善研究的流程

对研究进展提出建议

负责人员工作内容的记录以及财务的报销

做被试的随机分配

团队3：项目干预团队的内容包括：

接受培训，掌握干预的方法

提供高质量的初始评估

提供符合研究设计的心理咨询服务

对心理咨询流程的匹配度做打分

对心理咨询的过程做督导和反馈

团队4：数据处理团队

对被试数据进行统计整理

清理数据

数据分析和解读

**研究介绍**
6.研究背景

1. 研究问题

2020年初爆发的新型冠状病毒肺炎疫情给全国乃至全世界都带来了极大的挑战。截止2020年3月19日，中国已经累积有8万多人确诊，国外也有累积5万余人确诊。新冠肺炎病毒的爆发让民众陷入恐慌。疫情期间首个大型精神卫生调查显示，35%的民众情绪应激反应明显，而因疫情而引发的焦虑、抑郁和精神分裂等症状等也十分值得关注（Qiu, Shen, Zhao, Wang, Xie,& Xu, 2020). 虽然在疫情期间, 儿童青少年所受的心理困扰程度最小 （Qiu et al., 2020），但不同的研究都表明，儿童青少年在灾后会出现不同程度的心理症状，包括急性应激创伤、焦虑、抑郁、创伤后应激障碍等 （Kar, 2009）。除此之外，疫情期间学校延迟开学 （Xinhua Net，2020），儿童青少年群体长期居家会因为减少和同辈、老师的交流，上网课注意力不集中，用眼过度等原因降低学习的效率，对学业产生一定的焦虑。这些由于疫情所导致的儿童青少年心理健康问题都不容小觑。

在疫情期间，北京师范大学心理学部联合北京师范大学学生心理咨询与服务中心紧急筹备开通的心理支持热线和网络辅导目前已为6000余人提供了专业的心理服务。在6000余人中，儿童青少年占总数的5%；其中高中生占儿童青少年总数的68%，初中生占17%。求助的青少年主要体现出基于疫情的焦虑情绪，同时表达了希望能学习有效的方法来改善当下的状况。基于多项研究证明，短程焦点心理咨询的方法可以在短期内对青少年焦虑提供一定程度的调节（Creswell et al.,2017; Franklin, Moore, & Hopson, 2008 ），同时短程焦点咨询对中国人有比较明显的干预效果（Kim, Franklin,Zhang, Liu, Qu,& Chen, 2015），因此本研究希望通过随机对照组的方式，来验证通过这一疗法采用网络的渠道，是否能有效调节青少年在疫情中产生的焦虑。

b)选择对比组的原因

由于疫情的紧迫性，本研究希望能尽快的找到合适的短程干预方式来帮助青少年调节焦虑，因此干预时间只计划持续2周。为了减少其他基于咨询师的内部差异而导致的咨询方式差异，本研究采用干预组和等待组的方式进行随机对照试验。经过等待组的被试在完成一个月的追踪测后还能够得到心理咨询的支持，整体受影响相对较小。

7.研究目的

本研究旨在借鉴短程焦点的干预模型，通过以资源优势为基础的方为在新冠肺炎疫情中有焦虑的表现青少年提供网络短程干预。本研究首要目的是了解通过2-4次的心理咨询，青少年的焦虑的感受是否会有变化。次要目的是探索该方法是否对青少年的抑郁状态和应对困境的方式产生了短期的改变。为验证该研究方案的有效性，拟招募至少76位在疫情期间到中重度焦虑，无即刻自杀自伤风险，无过往精神疾病史的青少年，采取随机对照试验进行验证。最后该研究还希望了解来访者对短程焦点心理咨询服务的满意程度。

8.研究设计

该研究将采用随机对照试验、数据分析员单盲的方式来比较接受短程焦点干预的小组和等待组的成员在2周干预期后其焦虑程度的变化。同时，本研究设计了解在1个月后进行数据的重测随访，了解咨询干预有效性的维持程度。干预组和等待组的成员将以1：1的数量进行随机分配。

**研究方法：研究对象、干预方式、效果评估**

9.研究场地：

疫情期间，中国实行了严格的管控制度。几乎所有的学校都停学，学生们和家长大部分时间都处于居家的状态。因此本次研究工作全部通过网络平台来进行。包括研究个案的招募、研究被试的沟通、研究中干预的进行。招募信息都通过网络平台，例如微信公众号、微信朋友圈、微信群等进行发布。来访者会在报名后留下自己的联系方式（例如电话、邮箱、微信、qq）。工作人员会通过这些方式和被试进行面谈和咨询。

10.研究对象入组标准

a)入组标准：

1. 年龄在11-18岁的中学生，目前正在初高中就读；
2. 在新冠肺炎疫情期间表现出焦虑症状，且GAD-7 分数大于等于10分；
3. 自愿参与研究且得到监护人的知情同意；
4. 有稳定的网络环境和安静的场所来接受网络咨询。

b)排除标准：

1. 有2周以内的自杀意念和自杀计划；
2. 目前正在参加其他心理咨询的；
3. 目前正在使用精神科药物；
4. 有严重的精神病性障碍诊断；
5. 不愿意让其监护人知晓并签署知情同意的；

11. 干预方式

通过入组标准的被试将被随机分配至干预组或是等待组。进入短程焦点治疗干预组的被试，会和咨询师沟通其咨询目标，并且按照SFBT的咨询流程进行一周1-2次，两周总共不超过4次的干预。所有参与干预项目的咨询师都接受了10小时的短程焦点治疗培训，并且通过了干预执行程度的考核。

如果在干预过程中，研究者发现来访者的议题并无法通过短程咨询的框架进行解决。咨询师会在来访者利益最大化的前提下和来访者进行沟通，澄清来访者的目标，并且表达短程咨询的局限性。如果来访者决定采用其他的治疗方法，咨询师会和来访者进行转介，同时来访者将退出该研究项目。

在研究的过程中，被试每完成一个阶段的研究就将得到一个阶段的被试费。被试费会通过三个不同的阶段发放。第一个阶段为结束初次评估确认入组后，被试和家长若都填完了对应的量表，则可以拿到30元被试费。当被试结束2周的咨询并且填写了相对应的量表后，被试将得到第二阶段的被试费120元。在被试完成1一个月的随访量表填写后，他们将得到第三阶段的被试费50元。在中途退出项目的被试将无法拿到该阶段及之后的被试费。

咨询师在咨询过程中将为了研究需要全程录音，并于结束后进行录音的逐字稿转录。转录之后，会有其他研究人员对其干预和设定流程的匹配性进行核对，已确保咨询师在按照短程焦点的框架进行干预。督导师会每周给咨询师进行一个2小时的团体督导，对被试个案的进展进行讨论，并且及时指出咨询师在干预过程中的问题，确保咨询符合研究的标准和框架。

12. 结论

a)首要结论的评估方式

焦虑状态问卷中文版（STAI-Y (C)）和广泛性焦虑量表（GAD-7）将作为首要结论的评估工具来衡量来访者的焦虑程度。研究者将通过对基线期数据和2周后干预组和等待组数据差异的前后对比来评估短程焦点解决的干预方式是否会在降低来访者的焦虑程度上出现临床意义上的显著区别。研究者还会通过评估1个月后的追踪测数据来考察短程焦点治疗对焦虑改善效果的维持性。

b）次要结论的评估方式

病人健康状况问卷青少年版 （PHQ-9 A）和中学生应对方式量表 （CSS）是量个次要结论的评估方式。由于焦虑和抑郁的共病程度很高，被试很有可能在焦虑的同时伴随抑郁。同时，青少年阶段的不适应性抗压方式也会影响其情绪。研究者将通过对被试的抑郁程度和应对方式两个方面，考察短程焦点的咨询方式是否会改变来访者的抑郁程度，提升其有效应对的方式。

c）其他结论的评估方式

本研究还使用了来访者满意度问卷来评估被试在结束干预之后对该干预方式的评价，以了解该干预方式是否让来访者满意。

13. 参与时间轴

**
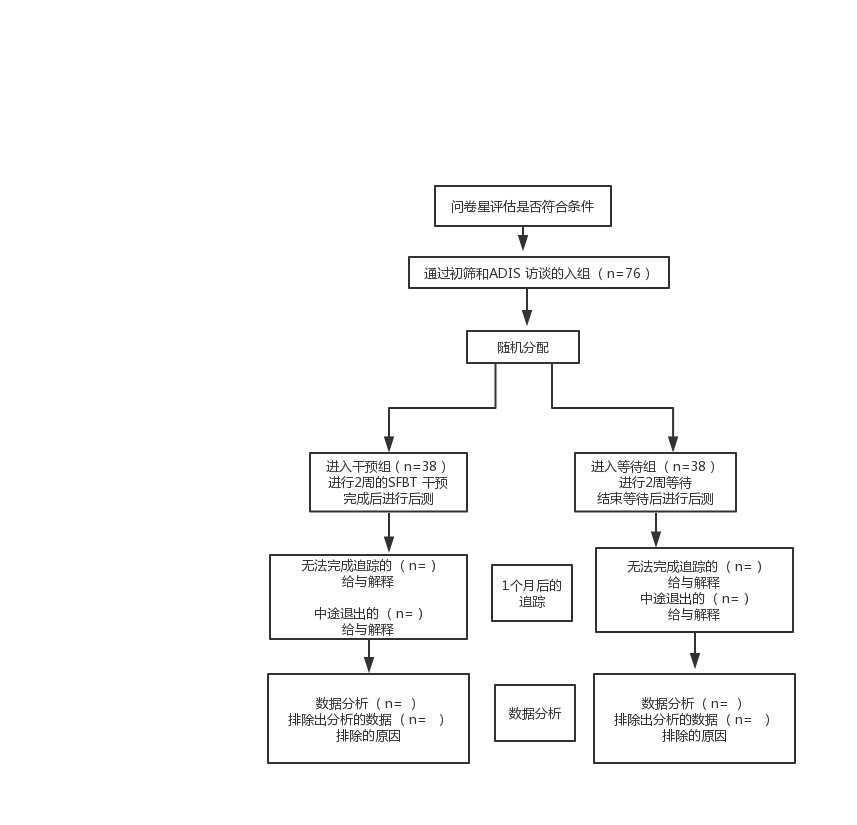
**

14. 样本量

因为前人没有做过类似的研究，因此无法基于前人的文献来进行样本量计算。经过计算发现两个组的样本共达到68例时可以达到中到大的效应量。预计在研究过程中有10%的脱落率，最后的样本量定位76。按照1比1的分配方式计算，每个组的入组人数为36人。

15. 招募方式

本研究通过公共渠道和私人渠道两种方式进行招募。公共的平台包括北京师范大学心理学部公众号“京师心理大学堂”及北京师范大学心理学部心理健康服务中的公众号。私人渠道包括通过把招募信息发给和青少年群体有密切交集的老师、家长等方式。招募内容通过发放招募海报、发送和招募相关的文章等对该研究进行介绍。符合招募中介绍内容的被试可以通过扫描海报上的二维码进行知情同意书的阅读。点击同意参与该项目才能开始做初始评估问卷。基本人口学信息也会通过初试的问卷进行收集。通过初步筛选，我们将满足入组标准的来访者纳入，剔除不符合被试要求的来访者。评估团队会对初步进入的个案进行详细的ADIS结构化访谈，来明确来访者的焦虑程度，并且进一步确认来访者是否满足入组条件。确定入组的来访者进入下一阶段的评估和干预。

**研究方法：干预组的分配方式**

16. 分配

每一位进入研究的被试都被分配到一个对应的研究序号。研究者通过网络上的随机数字产生工具将被试的编号范围输入该工具中。通过1：1的比例，随机将抽取到数字的被试分配进入干预组。剩余没有被抽取到的被试则进入等待组。

17. 盲法

由于本研究涉及干预组和等待组，因此参与项目的咨询师和研究助理都无法被盲。参与本次研究的初筛评估人员和数据收集员并不了解被试及其对应的研究编号，因此处于被盲的状态。

**研究方法： 数据收集，数据管理，数据分析**

18. 数据收集方法

数据评估和收集方法：

背景信息筛查

研究招募信息将通过问卷的形式，采用网络渠道发放给适龄的青少年。青少年需要点击同意参与研究的知情同意方可进入背景信息的填写。 收集的原始信息包括：姓名、年龄、性别、年级、所在地、联系方式、紧急联系人姓名和联系方式、是否有精神病性诊断、当前的主要困扰、是否当前接受心理咨询、是否在近两周有自杀自伤意念和行为、是否有接受网络咨询的条件、以及对咨询的期待。

在问卷星中，来访者还将填写GAD-7 和 PHQ-9 量表来评估其当下的焦虑和抑郁状态，作为是否能参与研究的筛选标准。

ADIS 面谈评估

在来访者符合最初的筛选标准后，访谈员将和来访者进行沟通，并且确认其监护人的知情同意。 若被试表示不愿意让其监护人知晓则无法进入研究的被试库。若来访者同意监护人签署知情同意书，则对来访者进行ADIS 面谈评估，来确定来访者是否有其他的精神病性诊断。 同时了解来访者的焦虑类型。

基线测量

来访者需要填写的测量内容包括：焦虑状态问卷中文版（STAI-Y (C)）以了解青少年当下的焦虑状态程度；病人健康状况问卷青少年版 （PHQ-9 A）以了解青少年的抑郁程度；中学生应对方式量表 （CSS） 以了解青少年当下应对挑战主要采用的方式。

来访者的父母同时需要填写：斯宾思儿童焦虑量表-家长版来判断来访者的焦虑情况是否和ADIS 中评估的一致；以及使用病人健康状况问卷问卷青少年版家长自我报告 （PHQ-9：Modified for Teens parent report ）来了解家长眼中的青少年抑郁程度。

咨询后测量

来访者需要填写基线测量中的广泛性焦虑障碍量表（GAD-7）；焦虑状态问卷中文版（STAI-Y (C)）；病人健康状况问卷青少年版 （PHQ-9 A）；中学生应对方式量表 （CSS）。同时增加一个来访者满意度问卷来评估参与干预组的来访者对参与咨询的满意程度。

来访者父母需要通过填写基线期的问卷，包括斯宾思儿童焦虑量表-家长版和病人健康状况问卷问卷青少年版家长自我报告。

追踪测量

一个月后，所有来访者和其父母都将重新填写基线期的所有量表。包括来访者填写的广泛性焦虑障碍量表（GAD-7），焦虑状态问卷中文版（STAI-Y (C)）；病人健康状况问卷青少年版 （PHQ-9 A）；中学生应对方式量表 （CSS）。以及家长填写的斯宾思儿童焦虑量表-家长版和病人健康状况问卷问卷青少年版家长自我报告。

| 措施 | 管理时间（分钟） | 回应者 | 时间点 | | | |
| --- | --- | --- | --- | --- | --- | --- |
|  |  |  | S | T0 | T1 | T2 |
| **招募期的初筛** | | | | | | |
| GAD-7 | 2min | 孩子 | x | x | x | x |
| PHQ-9 | 3min | 孩子 | x | x |  |  |
| **青少年焦虑测量** | | | | | | |
| *焦虑面谈时间表-儿童（ADIS-C）* | 50min | 孩子 | x |  |  |  |
| STAI-Y (C） | 5min | 孩子 |  | x | x | x |
| 斯宾思儿童焦虑量表-家长版 | 10min | 家长 |  | x | x | x |
| **其他测量维度** | | | | | | |
| 病人健康状况问卷青少年版（PHQ-9 A） | 3min | 孩子 |  | x | x | x |
| 病人健康状况问卷问卷青少年版家长自我报告 （PHQ-9：Modified for Teens parent report ） | 3min | 家长 |  | x | x | x |
| 中学生应对方式量表 (CSS) | 15min | 孩子 |  | x | x | x |
| **评价量表** | | | | | | |
| 来访者满意度问卷 (CSQ-8) | 5min | 孩子 |  |  | x |  |
| **注：S= Screening T0=Baseline，T1= After treatment, T2= 1 Month followup** | | | | | | |

被试在参与项目之前，会了解整个项目的进程和参与的时间流程。在评估被试的参与意向以及其家长的配合意向后，被试进入到研究得了流程中去。在研究进行的过程中，被试会在不同阶段收到该阶段的被试费已作为鼓励其继续留在项目中的方式之一。被试还可以通过和科研小助手的沟通来解答其中的困惑，帮助其顺利参与到该项研究中去。

19. 数据管理

项目组申请了专门的问卷星账号，所有的数据都通过问卷星进行收集。该账号只能被研究主要负责人保管，其他人均无法接触到原始数据。研究主要负责人会将被试个人身份信息进行加密保管，该身份信息只分享给和被试进行沟通的科研小助手。数据分析员只能看到被试的编号及其量表的分数，不能看到被试所属的组别。

在完成所有的数据收集后，数据分析员会将数据进行清理，并且将其放入SPSS中进行进一步的数据分析。

20. 统计方法

本研究将通过SPSS 版本26来进行数据分析。在干预组和等待组的被试人口学信息中，成对样本T检验会被用来比较连续数值，而卡方检验(Chi-square test)会被用来测量质性变量。广义线性模型及重复测验会被用来比较干预组和等待组被试的数据。在比较过程中，时间（前测和后测）是个体比较的因素，而不同的组别（干预组和等待组）是被试间的比较因素。GAD-7, STAI-Y(C), PHQ-9, CSS中的不同分量表数据都会被进行前后的对比。p<.05 是判断数据结果师范为临床显著的标准。治疗意向分析法中的最后观察值推估将被用来分析退出研究的数据。

**研究方法：监管**

21.数据监管

数据将由北京师范大学心理学部伦理审核委员会监管。若在研究过程中有任何违规的行为，伦理委员会将对数据进行调查。

22.伤害

在研究过程中，研究团队将把被试的利益放在第一位，本着善行、无伤害的伦理原则质性本研究。被试在研究过程中若有任何议题发生都可以随时停止研究。在干预组的被试若对干预所采用的咨询方案有不适应之处，则可以通过转换咨询方式的方式来找到更适合其需求的干预。等待组的被试若有咨询需求无法进行等待也能得到合理的转介。完成等待期的被试会被提供2-4次免费咨询的机会，帮助其降低焦虑。

23.审计

本研究将按照学校严格的审计流程接收其检验。若中间出现任何问题，则研究项目组会提交对应的材料来回答审计中提出的问题。

**伦理和数据发布**

24.研究的伦理审核

北京师范大学心理学部伦理委员会与2020年3月13日对本研究进行伦理审核并予以通过。研究审核编号为202003130012。

25.研究计划书的修订

若在研究执行过程中，研究者需要修改研究计划中中的任何部分，主要研究总负责人都将做完善的记录并且汇报给研究团队、被试、中国临床试验注册中心、及北京师范大学心理学部伦理委员会。

26.知情同意

所有参与该研究的被试及其法定监护人都签署了研究知情同意书几咨询知情同意书（见附录）。由于新冠肺炎疫情期间，中国实施了严格的隔离政策，因此所有工作都通过网络进行，知情同意书也通过网络的方式进行签署。

27. 保密原则

所有被试的身份信息、前后测数据信息及心理咨询过程信息都将被严格保密。其中身份信息只有研究者项目负责人及两位研究小助手才能获得。前后测的数据信息只有研究项目负责人及数据分析员能获得。心理咨询的过程记录及录音只有咨询师本人及督导师能获得。获取的信息都将通过双重密码保护的方式被严格保管。

28. 利益澄清

本研究主要负责人和该项目之间没有利益关系。基金会的支持经费均作为被试费、咨询费、研究人员劳务费、外聘专家咨询费等方式发放。

29.数据的可触达性

本研究的数据将被保管在北京师范大学心理学部心理健康服务中心的数据库内。完成研究并发表后的6个月后该数据可被分享。若需要得到数据，[请发邮件至chenshitao@bnu.edu.cn](mailto:请发邮件至chenshitao@bnu.edu.cn)

30.研究结束后的照顾

对于研究结束后仍然有咨询需求的被试，研究小助手将提供其他心理咨询的转介渠道。

31．发表规则

该研究项目最终将计划被写成论文并做发表。参与该项目的实施并且对写论文有贡献的参与者将根据投入的程度得到相应的著作权。

**附录**

32. 知情同意书

**青少年心理咨询研究知情同意书**

感谢你报名参加北京师范大学心理学部心理健康服务中心陈师韬老师的《疫情期间青少年焦虑短程心理咨询有效性研究》。在研究正式开始之前， 请你仔细阅读以下细则：

1. 本研究项目题名为“疫情期间青少年焦虑短程心理咨询有效性研究”。该研究旨在通过提供3至4次短程心理咨询干预，来探究该咨询方式对降低疫情期间青少年焦虑程度的有效性。

2. 本研究的基本流程为：

（1） 筛选

在你和你的家长同意并签署该研究知情同意书，同时通过我们的问卷初筛后，你会首先参与一个时长为40-50分钟的访谈。 我们会根据访谈结果来评估你是否适合进入该研究项目。

（2）初始评估阶段

i) 若你符合入组标准，项目工作人员会和你取得联系，并且请你和你的父母分别填写一些初始量表。完成量表填写后，你将获得30元的被试参与费作为对你的感谢。

ii) 若你不符合入组标准，项目工作人员会根据你的情况为你提供其他心理健康服务资源的推荐。

（3）干预阶段

i) 在筛选确认入组后，你将会被随机分配到干预组或者等待组。若你参与到了干预组，咨询师将与你沟通咨询使用的平台，并且为你提供2-4次短程心理咨询服务。每次咨询在50 分钟左右，干预持续2周，每周1-2次。如果你被随机分配到了等待组，你会需要等待两周的时间。若在两周的等待期，你有强烈的需要咨询的需求，请和我们的工作人员联系，我们可以随时为你提供咨询的转介渠道。在等待期间开始咨询意味着你将退出我们的研究项目。

ii) 干预组和等待组的被试都将在结束两周的咨询或等待后再次填写一些问卷。你和家人完成所有的问卷填写后，你将获得120元的被试费。

（4）后测阶段

在完成上一轮的问卷评估后的一个月，工作人员会再次和你联系，并且邀请你和你父母通过网络再次填写一系列测评问卷。在你和父母认真填写完成所有的问卷后将得到50元的被试费。 如果等待组的被试届时有咨询意向，我们将为你提供2-4次的免费心理咨询服务。

3. 本研究中的心理咨询全程由北京师范大学临床与咨询方向接受过临床实践训练并通过考核的研究生提供。研究生会接受陈师韬老师的严格培训和督导。

4. 你在本研究中所接受的心理咨询为全程免费。完成整个项目可以总共获得200元的被试费报酬。报酬会在你结束该阶段的研究后，于5个工作日内发至你的支付宝或者微信钱包中。

5. 经过你的书面同意后，为了确保咨询的质量，我们的谈话过程将会被录音。录音资料的查看范围仅限于咨询师本人、督导师、以及该研究项目组的其他咨询师。所有收听录音者均承诺对相关内容严格保密，并签订保密协议，所有内容仅用于学术讨论、督导和研究，绝不对外泄露。

6. 经过你的书面同意后，咨询师将会根据咨询过程和咨询效果完成研究论文。在论文中将抹去你个人的身份可识别信息。你对此项研究结果享有知情权，如你想了解，可与陈师韬老师联系。联系方式为：11132018314@bnu.edu.cn

7. 为了保证咨询效果和研究进程，我们希望你能尽量保证稳定参与心理咨询，并完成整个干预过程。但若咨询或研究过程令你感到不适，你有权随时提出中止参与本研究。未能完成的研究部分将无法得到相对应的被试费。

8. 如你被确定进入研究项目，还需签署北京师范大学心理健康服务中心心理咨询知情同意书，关于心理咨询过程更详细的设置，以中心心理咨询知情同意书为准。

本知情同意书一式两份，自签订之日起生效，请妥善保管。如你和你的监护人（父母）已充分阅读并理解以上 内容，同意在如上所述的设置下作为心理咨询来访者参与本研究，请签字。

签字：

日期：

**网络咨询知情同意书**

欢迎您参与北京师范大学心理学部心理健康服务中心的青少年心理咨询。为明晰您与咨询师各自的权利与责任，在咨询开始前，请您认真阅读以下内容：

一、本协议为北师大心理学部心理健康服务中心知情同意书的网络版，仅适用于本次新冠肺炎疫情期间进行的网络咨询。

二、本协议中的咨询双方应均为完全行为能力人，未成年人应委托监护人履行协议认同手续。本协议不针对无行为能力人。

三、根据《中华人民共和国精神卫生法》等法律法规，本中心不提供心理治疗及精神障碍诊断、治疗等相关服务；如果咨询过程中，咨询师发现您可能存在上述情况，将建议到法定医疗机构就诊。

三、心理咨询是来访者与咨询师共同工作的过程，其效果取决于双方配合等多种因素。本中心咨询师均遵循相关法律规定和工作伦理，使用有临床研究基础、广泛使用并证实有效的流派技术，但也无法承诺必将解决您的问题。请您与咨询师一齐努力，制定并达成合理的咨询目标。

提供咨询的咨询师为我部经过一年系统化专业培养的专硕在读研究生及老师，通过考核后在督导师的监督保障下提供咨询服务。督导师为中心聘用的专业资深的专职督导师。

四、关于保密性 通常情况，只有您的咨询师才能接触到您相关的咨询信息，任何涉及可能显示您个人身份、咨询情况的信息，都将受到严格保护，包括在下述情况：

1. 根据咨询设置，为保证咨询效果，研究过程中发生的所有咨询须接受督导。您咨询师的督导师及其参加同一督导的咨询师，可能会获知您的咨询情况，但涉及您个人身份的相关信息将受到严格保密；同时他们将签署保密协议，不外泄包括您个人身份、咨询情况在内的任何咨询信息。

2. 鉴于北师大心理学部教学科研机构的身份及本次科研的性质，咨询师会将咨询过程用于科研，也可能会在教学科研过程中引用或讨论除可能显示您个人身份信息之外的咨询情况；但此举亦需经您书面同意后方可进行。

3. 经双方协商一致，您书面同意的情况。

保密例外

1. 经咨询师评估，发现有精神疾病倾向，可能对您自身或他人的生命安全构成严重危害（包括儿童侵害）的情况，咨询师有权利直接联系对您及他人生命安全负责的相关人员或相关机构。

2. 司法机构强制要求配合。

六、录音录像

为了保障本研究过程中咨询师按照所要求的咨询方式进行干预，咨询师将全程录音。咨询师承诺录音文件严格保密，并承担录音录像文件的保密保管责任。

七、咨询设置

1. 遵照心理咨询设置要求，咨询通常一周一次，每次咨询时间为50分钟。请按约定的时间登录网络账号。如您迟到，咨询仍在原定时间结束；如咨询师迟到，将补足迟到的咨询时间。

2. 预/续约成功后，您和咨询师双方均可提出更改咨询。如您有要事与咨询冲突，请务必在咨询前24小时提出更改；如您在咨询前24小时之内更改咨询，您只能将咨询更改至后天或之后的时间。

3. 鉴于研究的性质，本咨询研究将使用短程心理咨询的模式，由来访者与咨询师共同确立短期内能够达成的咨询目标，咨询过程也将聚焦于实现这些咨询目标，咨询持续3次。如您需要长程心理咨询，中心会为您提供相应的转介信息。原则上，本阶段三次咨询结束后，来访者将不再转介至本中心继续工作。

4. 如您与第一位咨询师在工作初期觉得不匹配，可以转介项目组其他咨询师（咨询次数以与第二位咨询师工作次数为准），但仅能转介一次；您亦可寻求其他机构的转介信息。

5. 如您连续两次24小时内主动取消预约或爽约达到两次，视为放弃参与本次研究。项目组仅将为您结算已完成阶段的被试费。

6. 您和咨询师双方均有权在咨询过程中的任何时间提出终止咨询，但我们希望您务必和您的咨询师妥善商量此事，至少进行一次结束性会谈后，不再续约。

7. 请您实名签署该协议；并承诺在接受中心服务期间，只同时与一位咨询师工作；若发现您可能同时预约了其他咨询师，中心将视情况决定是否再向您提供咨询服务。

八、其他

本协议具有同等法律效力，来访者勾选签字后生效，可在提交前使用预览模式打印保留（PC端）/或截图保存（移动端），请妥善保管。如来访者是未成年人，须同时由监护人签字；如两人以上一同前来的成年来访者，请同时签署。在来访者退出咨询后，协议终止。

来访者签名：

咨询师签名：

日期：

**参考文献**

Creswell, C., Violato, M., Fairbanks, H., White, E., Parkinson, M., Abitabile, G., ... &

Cooper, P. J. (2017). Clinical outcomes and cost-effectiveness of brief guided parent-delivered cognitive behavioural therapy and solution-focused brief therapy for treatment of childhood anxiety disorders: a randomised controlled trial. *The Lancet Psychiatry, 4*(7), 529-539.

Franklin, C., Moore, K., & Hopson, L. (2008). Effectiveness of solution-focused brief

therapy in a school setting. *Children & Schools, 30*(1), 15-26.

Johnny S. Kim, Cynthia Franklin, Yingping Zhang, Xuanwen Liu, Yuanzhou Qu & Hong

Chen (2015) Solution-Focused Brief Therapy in China: A Meta-Analysis, *Journal of Ethnic & Cultural Diversity in Social Work, 24*(3), 187-201, DOI: 10.1080/15313204.2014.991983

Qiu J, Shen B, Zhao M, et alA nationwide survey of psychological distress among

Chinese people in the COVID-19 epidemic: implications and policy recommendations. *General Psychiatry,* 2020:33:e100213. doi: 10.1136/gpsych-2020-100213

Xinhua Net (Feb 26, 2020). China Focus: Schools start online courses as epidemic

control postpones new semester. 2020. <http://www.xinhuanet.com/english/2020->02/17/c_138792006.htm.

Kar N. Psychological impact of disasters on children: review of assessment and

interventions. *World Journal of Pediatric, 2009,5*(1), 5-11.
